# In the eye of the storm: impact of COVID-19 pandemic on admission patterns to paediatric intensive care units in the UK and Eire

**DOI:** 10.1186/s13054-021-03779-z

**Published:** 2021-11-17

**Authors:** Hari Krishnan Kanthimathinathan, Hannah Buckley, Peter J. Davis, Richard G. Feltbower, Caroline Lamming, Lee Norman, Lyn Palmer, Mark J. Peters, Adrian Plunkett, Padmanabhan Ramnarayan, Barnaby R. Scholefield, Elizabeth S. Draper

**Affiliations:** 1 Paediatric Intensive Care Unit, Birmingham Women’s and Children’s NHS Foundation Trust, Birmingham, UK; 2 Birmingham Acute Care Research Group, Institute of Inflammation and Ageing, University of Birmingham, Birmingham, UK; 3 Leeds Institute for Data Analytics, University of Leeds, Leeds, UK; 4 Paediatric Intensive Care Unit, Bristol Royal Hospital for Children, Bristol, UK; 5 Department of Health Sciences, George Davies Centre, College of Life Sciences, University of Leicester, Leicester, UK; 6 Paediatric Intensive Care, Great Ormond Street Hospital NHS Foundation Trust, NIHR Biomedical Research Centre, London, UK; 7 University College London Great Ormond Street Institute of Child Health, London, UK; 8 Children’s Acute Transport Service, Great Ormond Street Hospital NHS Foundation Trust, NIHR Biomedical Centre, London, UK

**Keywords:** COVID19, Paediatric intensive care unit, Admission patterns, Case mix

## Abstract

**Background:**

The coronavirus disease-19 (COVID-19) pandemic had a relatively minimal direct impact on critical illness in children compared to adults. However, children and paediatric intensive care units (PICUs) were affected indirectly. We analysed the impact of the pandemic on PICU admission patterns and patient characteristics in the UK and Ireland.

**Methods:**

We performed a retrospective cohort study of all admissions to PICUs in children < 18 years during Jan–Dec 2020, using data collected from 32 PICUs via a central database (PICANet). Admission patterns, case-mix, resource use, and outcomes were compared with the four preceding years (2016–2019) based on the date of admission.

**Results:**

There were 16,941 admissions in 2020 compared to an annual average of 20,643 (range 20,340–20,868) from 2016 to 2019. During 2020, there was a reduction in all PICU admissions (18%), unplanned admissions (20%), planned admissions (15%), and bed days (25%). There was a 41% reduction in respiratory admissions, and a 60% reduction in children admitted with bronchiolitis but an 84% increase in admissions for diabetic ketoacidosis during 2020 compared to the previous years. There were 420 admissions (2.4%) with either PIMS-TS or COVID-19 during 2020. Age and sex adjusted prevalence of unplanned PICU admission reduced from 79.7 (2016–2019) to 63.1 per 100,000 in 2020. Median probability of death [1.2 (0.5–3.4) vs. 1.2 (0.5–3.4) %], length of stay [2.3 (1.0–5.5) vs. 2.4 (1.0–5.7) days] and mortality rates [3.4 vs. 3.6%, (risk-adjusted OR 1.00 [0.91–1.11, *p* = 0.93])] were similar between 2016–2019 and 2020. There were 106 fewer in-PICU deaths in 2020 (*n* = 605) compared with 2016–2019 (*n* = 711).

**Conclusions:**

The use of a high-quality international database allowed robust comparisons between admission data prior to and during the COVID-19 pandemic. A significant reduction in prevalence of unplanned admissions, respiratory diseases, and fewer child deaths in PICU observed may be related to the targeted COVID-19 public health interventions during the pandemic. However, analysis of wider and longer-term societal impact of the pandemic and public health interventions on physical and mental health of children is required.

## Background

Coronavirus disease (COVID-19) caused by severe acute respiratory syndrome coronavirus 2 (SARS-CoV-2) during this global pandemic has been rare in children compared to adults [1]. The number of children requiring paediatric intensive care unit (PICU) admission with either acute COVID-19, or related conditions such as the novel post-covid inflammatory syndrome referred to as paediatric inflammatory multisystem syndrome temporally associated with SARS-CoV-2 (PIMS-TS) or multisystem inflammatory syndrome in children (MIS-C), was small in comparison with the number of adults requiring critical care admission following SARS-CoV-2 infection [2, 3]. Nonetheless, the pandemic affected critically ill children and PICUs in many other ways [4].

Several PICUs were either entirely or partially repurposed to provide adult critical care [5]. During the crisis most institutions reduced their planned surgical activity. However, changes to the pattern of emergency admissions to PICU have also been observed [6–8]. To date, however, there have been no national population–level reports describing the impact of the pandemic and the ‘lockdown’ periods during the whole of 2020 on critical care admissions in children. We aimed to analyse whether PICU activity in 2020 differed from the previous years across the whole of theUK and Republic of Ireland (RoI) using the paediatric intensive care audit network (PICANet) database. In particular, we aimed to analyse the nature and magnitude of differences in admissions, bed-activity, case-mix, interventions, and outcomes of PICU admissions during 2020, compared with preceding years.

## Methods

We performed a retrospective analysis of routinely collected information for all admissions to PICUs in children less than 18 years of age from all UK and RoI PICUs (*n* = 32). Admission patterns, case-mix, resource use and outcomes during the initial pandemic year (2020) were compared with the four preceding years (2016–2019), based on the date of admission.

### Data collection

Since 2002, PICANet has collected, analysed and reported information related to admissions from PICUs in England and Wales. Data collection was expanded over subsequent years to include PICUs in Scotland, Northern Ireland (NI) and the Republic of Ireland [9]. PICANet has a validated comprehensive database containing clinical and demographical information on over 300,000 PICU admissions and over 2 million bed-days. Data quality is maintained through central and local data validation as well as regular site visits. The admission dataset includes information related to patient demographics, diagnoses, interventions included in the paediatric critical care minimum dataset, and vital status at discharge from PICU, as well as severity of illness variables at the time of admission for calculation of the Paediatric Index of Mortality-3 (PIM3) score [10].

Primary diagnoses, comorbidities and procedures are coded using Read Clinical terms version 3 codes. Diagnostic category was assigned on the basis of the primary diagnosis submitted to PICANet by the clinicians from the relevant PICUs. Specific diagnoses indicating the main reason for admission for conditions such as asthma, bronchiolitis and diabetic ketoacidosis (DKA) were extracted from the PIM3 part of the dataset. A planned admission, as defined by PICANet data manual available at www.picanet.org.uk, is an admission that could have been delayed for more than 24 h without risk or is a post-operative admission of which a PICU is aware of before the surgery begins. At the start of the pandemic, a customised dataset was established to collect information about all children admitted to PICUs with diagnoses of COVID-19 and/or PIMS-TS. Age–sex adjusted prevalence estimates used mid-year population estimates for the most recent year of publicly available age–sex population data for each country at the time of analysis (May 2021) [11, 12].

### Statistical analysis

Descriptive data are reported as numbers and percentage for categorical variables and medians with interquartile ranges (IQR) and mean ± standard deviations for continuous variables. Data collected from 2020 activity were compared with the 2016–2019 (‘pre-pandemic’) period. Absolute differences in proportion of patients (with 95% confidence intervals), relative to overall number of admissions, as well as relative differences between 2020 compared with 2016–2019 mean numbers are reported. In-PICU mortality was assessed using a multilevel logistic regression model to account for any potential clustering at the PICU level with adjustment made for PIM3 predicted probability of death. Statistical process control charts were used, if required, to highlight special cause variation, if any [13].

### Ethics

Processing of personally identifiable data for the purposes of service evaluation, audit and research was approved by the Patient Information Advisory Group (now the Health Research Authority Confidentiality Advisory Group) in 2002 under Section 60 of the Health and Social Care Act (subsequently Section 251 of the National Health Service Act 2006) (reference: PIAG 4-07(c) 2002). This was amended and approved specifically to collect additional data relating to COVID-19 for confirmed and suspected cases.

## Results

### Admissions and bed activity

**Fig. 1:**
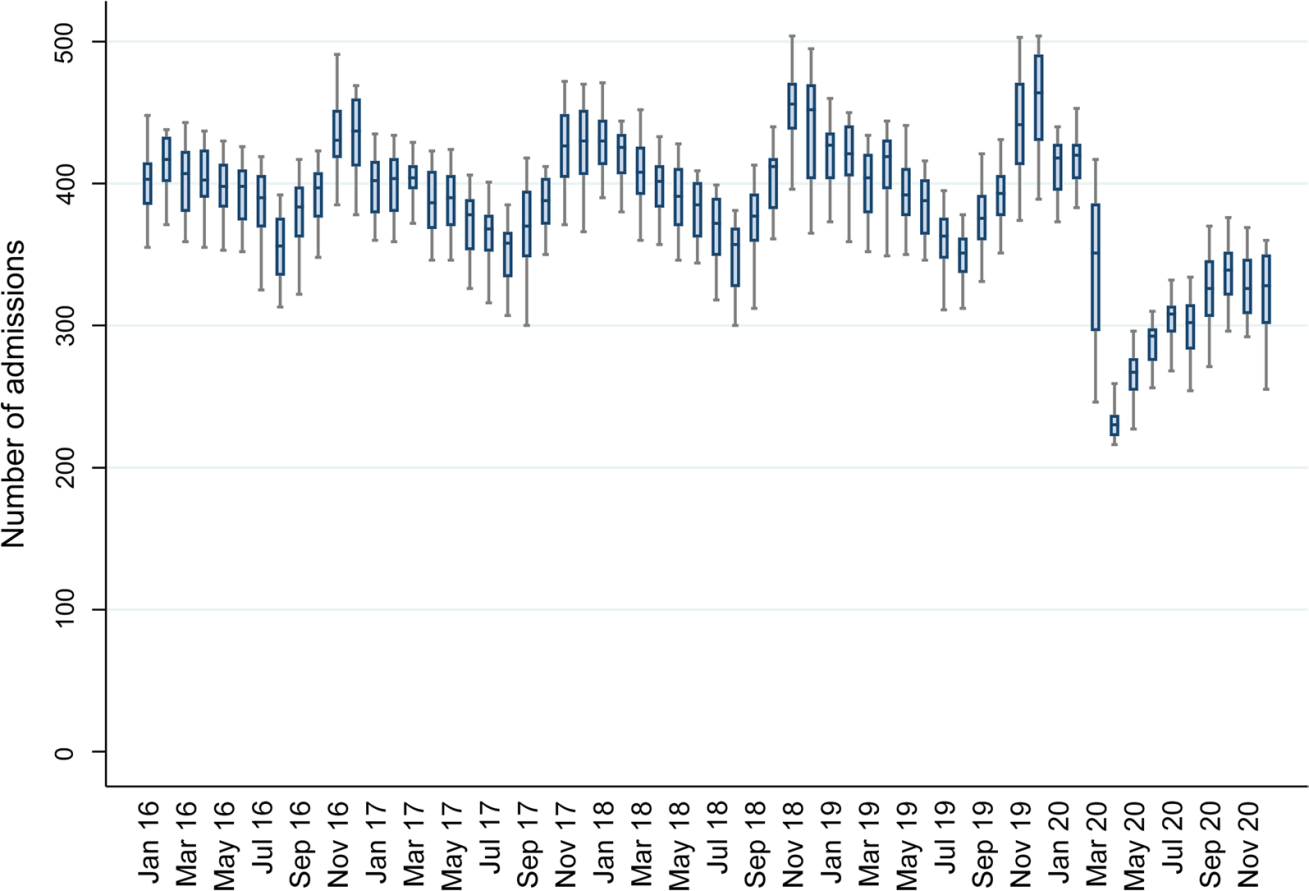
Boxplot of monthly PICU bed activity during the study period

**Fig. 2:**
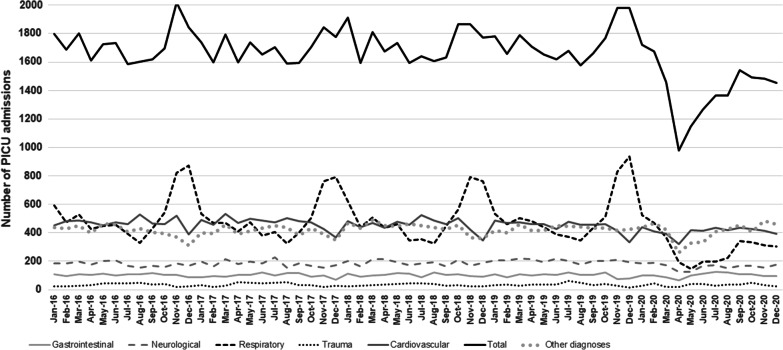
Admission trends in different primary diagnostic categories during the study period

During 2020, only 16,941 PICU admissions were observed compared to a mean of 20,643 (range 20,340–20,868) per year over the period 2016–2019. This represents an 18% reduction in PICU admission numbers in 2020 compared to the mean of the pre-pandemic period (Table 1). There were fewer PICU bed-days in 2020 with 109,216 bed-days recorded compared to a mean of 145,168 per year (range 140,836–147,985) during 2016–2019, representing a 25% reduction. The magnitude of reduction in PICU bed-days varied between the countries, with a 12% reduction in RoI to 31% in Scotland. Notably, there were 32% fewer PICU admissions during the first period of ‘lockdown’ (April–June 2020) [14], and 20% fewer admissions during the period October to December 2020 compared to the mean number in the same months for 2016–2019. The impact was not equally distributed across PICU activity: for example, there was a greater reduction in unplanned (20%) than planned admissions (15%) in 2020 compared with 2016–2019. A sharp reduction in admissions was observed in March 2020 with the nadir of PICU admissions occurring in April 2020 (Fig. 1). This was followed by a slow progressive increase in the number of admissions over the next few months. Notably, however, the typical winter peak in PICU admissions observed in 2016–2019 did not occur in 2020 (Fig. 2). Age–sex adjusted prevalence per year of unplanned PICU admissions fell from 79.7 (2016–2019) to 63.1 per 100,000 in 2020. Some variation in unplanned admissions was observed between countries in both 2020 (range 37.8 in NI to 68.3 in Scotland) and the 2016–2019 period: (range 74.7 in ROI to 91.5 in NI) with the reduction in prevalence ranging from 14.0 admissions per 100,000 in ROI to 53.7 admissions per 100,000 in NI.

**Table 1:** Differences in PICU admissions, bed-days and diagnostic categories between 2020 and 2016–2019

	2016–2019 aggregate	2020	Comparison (2020 vs. 2016–2019)	
	Mean (range)	*n*	Absolute difference	Relative difference (%)
*Number of PICU admissions*				
January–March	5239 (5134–5319)	4846	− 393	7.5
April–June	5008 (4982–5059)	3393	− 1615	32.3
July–September	4869 (4788–4921)	4270	− 599	12.3
October–December	5527 (5326–5733)	4432	− 1095	19.8
Annual	20,643 (20,340–20,868)	16,941	− 3702	17.9
*PICU bed days*				
January–March	37,541 (36,963–38,922)	31,122	− 6419	16.3
April–June	34,675 (32,823–35,965)	24,709	− 9966	28.7
July–September	33,593 (32,438–34,480)	28,851	− 4742	14.8
October–December	39,359 (32,248–40,980)	24,534	− 14,825	37.7
Annual	145,168 (140,836–147,985)	109,216	− 35,952	24.8

	Median (IQR)	Median (IQR)		Difference in medians
PIM3 predicted probability of death	1.2 (0.5–3.4)	1.2 (0.5–3.4)		0.0

	Mean *n* (%)	*n* (%)	Difference in proportions (95% CI)	Absolute difference (Relative difference, %)
*Admission type*				
Planned	8275 (40.1)	7065 (41.7)	1.6 (0.8 to 2.4)	− 1210 (− 14.6)
Unplanned	12,364 (59.9)	9862 (58.2)	− 1.7 (− 2.4 to 0.9)	− 2502 (− 20.2)
*Primary diagnosis/reason for PICU admission*				
Respiratory	6119 (29.6)	3601 (21.3)	− 8.3 (− 9.0 to − 7.6)	− 2518 (− 41.1)
Unplanned respiratory^*^	5117 (41.4)	2922 (29.6)	− 11.8 (− 12.8 to − 10.8)	− 2195 (− 42.9)
Trauma	419 (2.0)	394 (2.3)	0.3 ( 0.1 to 0.5)	− 25 (− 6.0)
Diabetic ketoacidosis	142 (0.7)	261 (1.5)	0.8 ( 0.6 to 1.0)	+ 119 (+ 83.8)
Asthma	399 (1.9)	239 (0.7)	− 1.2 (− 1.4 to − 1.1)	− 160 (− 40.1)
Bronchiolitis	1820 (8.8)	726 (4.3)	− 4.5 (− 4.9 to − 4.2)	− 1094 (− 60.1)
*Interventions*				
Invasive ventilation^†^	12,757 (61.8)	9999 (59.0)	− 2.8 (− 3.6 to − 2.0)	− 2758 (− 21.6)
Inhaled nitric oxide	808 (3.9)	641 (3.8)	− 0.1 (− 0.4 to 0.2)	− 167 (− 20.6)
CVVH	369 (1.8)	335 (2.0)	0.2 (0.0 to 0.4)	–34 (− 9.2)
ECLS	249 (1.2)	205 (1.2)	0.0 (− 0.2 to 0.2)	− 44 (–17.7)
*Outcomes*				
In-PICU mortality	711 (3.4)	605 (3.6)	− 0.2 (− 0.5 to 0.1)	–106 (− 14.9)
In-PICU mortality for respiratory admissions	134 (2.2%)	67 (1.9%)	0.3 (− 0.2 to 0.8)	− 67 (− 50.0)
Median length of stay (days)	2.3 (1.0–5.5)	2.4 (1.0–5.7)		0.1

*PIM* paediatric index of mortality, *CVVH* continuous veno-venous haemofiltration, *ECLS* extracorporeal life support, *denominator for percentage is number of unplanned admissions, ^†^on any day of PICU admission

### Respiratory

A large proportion of the difference in number of PICU admissions was accounted for by the respiratory diagnostic group comprising 68% of difference in PICU admissions in 2020 with 2518 fewer respiratory admissions (Fig. 2) compared with the mean for 2016–2019. Unsurprisingly this was mainly apparent in the youngest admissions: infants < 12 months of age saw a relative reduction of 45% in 2020 compared with 2016–2019 (1627 admissions in 2020 vs. a mean of 2964 admissions in 2016–2019) with a similar relative reduction seen in 1–4 year olds (44%, 1020 admissions in 2020 vs. mean of 1814 admissions in 2016–2019). The greatest reduction in respiratory admissions was for bronchiolitis with a relative reduction of 60% in admissions in 2020 compared with 2016–2019 (726 vs. mean of 1820). In particular, there were only 195 admissions for bronchiolitis between October and December 2020 compared with annual average of 1068 in same time period of 2016–2019, representing an 82% reduction.

### Trauma

In contrast to this 40% reduction in respiratory admissions in 2020 compared with respiratory admissions during 2016–2019, there was only a 6% relative reduction in trauma-related admissions observed during the pandemic.

### DKA

**Fig. 3:**
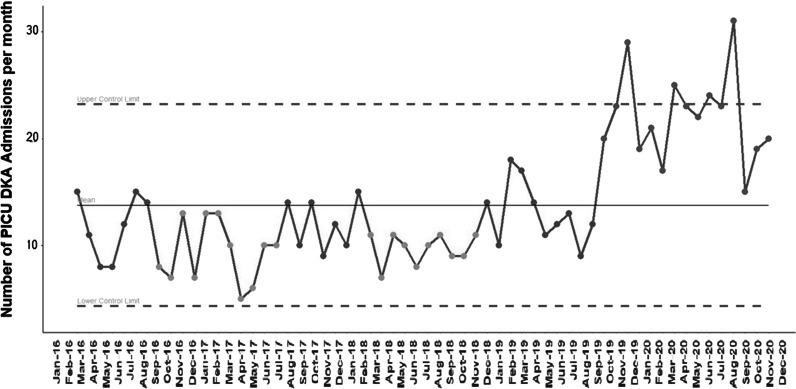
Control chart showing special cause variation in DKA admissions in PICU

In 2020, there were 261 PICU admissions for DKA accounting for 1.5% of all admissions compared to a mean of 142 per year (ranging from 123 to 188) in the years 2016–2019 (Fig. 3; Table 1), accounting for 0.7% of all admissions. Median (IQR) base-deficit of DKA patients at PICU admission during 2020 was 23 (16.6–26.5) compared to 22.5 (16.9–26.9) in 2016–2019. Median PICU length of stay for DKA admissions in 2020 (1.3 [0.8–2.2] days) was similar to 2016–2019 (1.2 [0.8–2.1] days).

### COVID-19/PIMS-TS

While there was a major impact of the pandemic on the overall numbers of admissions for PICU, the actual numbers of admissions of patients with COVID-19 and/or PIMS-TS only totalled 410 (2.4%) in 2020 with the highest numbers in April and May (77 and 106 admissions, respectively). This reflected the general population pandemic peaks for the UK.

### Post-operative admissions

There were 1271 fewer planned post-operative admissions to PICU in 2020 compared to the 2016–2019: mean 6633 versus 7904 (range 7763–7976). The main constituents of this reduction comprised admissions following non-cardiac surgery (726, 57.1%) and admissions following cardiac surgery (507, 39.9%) compared with the 2016–2019 average. PICU admissions after elective liver transplant remained stable with only a 0.5% reduction in activity through 2020 compared with the 2016–2019 average.

### PICU interventions

The proportion of admissions requiring invasive mechanical ventilation on any day of PICU admission in 2020 was statistically significantly lower than in 2016–2019 with a decrease of 2.8% (95% CI 2.0–3.6%). Although the absolute numbers were lower in 2020, the proportion of admissions requiring the highest level of PICU interventions such as inhaled nitric oxide, continuous venovenous haemofiltration (CVVH) and extracorporeal life support (ECLS) remained constant throughout both time periods.

### Outcomes

The PICU length of stay in 2020 was similar to the pre-pandemic period: median length of stay (LOS) 2.3 (1.0–5.7) days in 2020 versus 2.4 (1.0–5.5) days in 2016–2019. The in-PICU mortality rate in 2020 (3.6%) was similar to the previous years (3.4%). This was confirmed using a multilevel logistic regression model adjusting for PIM3-predicted probability of death (OR 1.00 [0.91–1.11, *p* = 0.93]). However, given the reduction in the number of PICU admissions during 2020, this resulted in a lower absolute number of child deaths compared to the mean number of deaths per year in 2016–2019 (605 in 2020 vs. 711 in 2016–2019). Of note, the in-PICU mortality rate for respiratory admissions was similar between the study periods [1.9% (2020) vs. 2.2% (2016–2019)], with an odds ratio of adjusted in-PICU mortality of 0.82 [0.63–1.06, *p* = 0.13].

## Discussion

During 2020, there were approximately 3700 fewer admissions, 36,000 fewer bed-days, and 100 fewer child deaths in PICU compared to the 2016–2019 average. There was a reduction in age–sex adjusted prevalence of unplanned PICU admission of 17 per 100,000 population in 2020 relative to the earlier period. The reduction in respiratory disease (in particular bronchiolitis) was the major contributor to the decrease in unplanned PICU admissions during 2020 with a striking absence of the expected winter peak. Conversely, there were significantly more admissions of patients with DKA compared to previous years. However, we did not observe an increase in morbidity or severity of illness using proxy measures such as probability of death using PIM3, length of PICU stay, advanced level of interventions and in-PICU mortality rates.

The virtual PICU systems (VPS) in the USA reported a 32% reduction in PICU admissions during April–June 2020 compared with 2017–2019 average admission figures for that quarter [7]. However, data from July–December 2020 were not reported. We observed an identical 32% reduction in PICU admissions during the same time period, followed by 12% and 20% reductions in July–September and October–December, respectively. PICU bed-days for admissions between October and December 2020 in this report were 38% lower than the comparable pre-pandemic period because of the striking absence of a winter peak. The VPS report observed that admission numbers for the time period between January and March 2020 were comparable to that of the previous years. However, we report a 7% reduction during the same time period relative to 2016–2019. The first cases of COVID-19 were reported in January 2020 in the UK, and in February 2020 in the RoI. Lockdown rules were imposed in the UK and RoI in the last week of March 2020 [15]. The ensuing reduction in bed activity that occurred in March 2020 is likely to have contributed to the difference. A report from Scotland that described data from their two PICUs, which are included in this dataset, also identified a similar reduction in PICU admissions with no substantial evidence of harm [6].

Reduction in respiratory disease accounted for the majority of the difference in the number of PICU admissions and bed-days. Reports from North and South America have also revealed a similar trend of a significant reduction of respiratory viral illnesses [7, 8]. The reasons are not entirely clear, although it may be related to the effectiveness of population-based non-pharmacological public health interventions. Several public health interventions were widely adopted in the UK and RoI during 2020 [14, 15]. This included ‘stay-at-home orders’, social distancing measures, mobility restrictions, school closures, mask wearing and shielding of vulnerable populations [14, 16]. Both the time periods with higher magnitude of reduction in PICU admissions and bed-days, i.e. April–June 2020 and October–December 2020, coincided with periods of national lockdown. Unlike April–June 2020, the stay-at-home orders were not accompanied by a national school closure during October–December 2020. However, there was a requirement for face covering, social distancing measures, better access to SARS-CoV-2 testing and isolation of symptomatic subjects. The direct impact of these public health interventions on COVID-19 has been reported [17, 18]. The 2003 epidemic of severe acute respiratory virus (SARS) triggered an examination of the effectiveness of non-pharmacological interventions on other illnesses. There is some evidence to support the effectiveness of some of the strategies used during this pandemic in the spread of respiratory infections including influenza, upper respiratory tract infections, and respiratory syncytial virus infections, with a notable effect in reducing secondary attack rates in young children [19, 20]. Given the reduction in the absolute number of child deaths in PICU reported in this study, as well as from other parts of the world [21, 22], it remains to be seen whether some of the public health measures such as social distancing, wearing masks or face coverings and frequent hand-washing continue to be widely adopted and remain sustained in the community. It is as yet unclear how the low levels of exposure to respiratory pathogens among infants and children during the pandemic will impact on future seasonal and inter-seasonal respiratory viral surges. However, reports from Australia and elsewhere have raised this as a concern [23].

An increase in DKA referrals was reported by one of the regional UK PICU transport services during the COVID-19 pandemic compared with previous years, despite a substantial reduction in overall referrals to the service [24]. A study from the USA also reported a significant increase in patients with diabetes mellitus in PICU, but perhaps not necessarily with DKA [7], while a report from across Canada identified a significant increase in both DKA and severe DKA presentations in new Type 1 diabetes mellitus [25]. Our analysis confirms the trend of a recent increase in PICU admissions for DKA. However, it is unclear whether an association exists with SARS-CoV-2 infection, given that the increase in DKA admissions was first observed prior to the pandemic (October–December 2019), but remained sustained at higher levels during the pandemic. Delayed diagnosis of diabetes resulting in presentation as a DKA admission was raised as a concern [26]. Comparisons of base deficit and length of stay of PICU admissions for DKA did not support an increase in severity of illness at PICU admission.

The VPS report described an increase in the proportion of trauma-related PICU admissions from 6.8% during April–June 2017–2019 to 9% of PICU admissions over the period April–June 2020, while the absolute numbers during this period in 2020 were lower than the previous years [7]. Our observations were similar, but with a smaller non-significant increase from 2% of all PICU admissions in 2016–2019 to 2.3% in 2020. Again, there were fewer absolute numbers of patients admitted with trauma during 2020 and the differences are perhaps explained by a larger reduction in the non-trauma admissions, such as respiratory infections.

Altered health seeking behaviour resulting in delayed presentation has been implicated as a potential concern during the pandemic [26]. Comparison of severity of illness on presentation to PICU during 2020 with data from the period 2016–2019, drawn from the analysis of the probability of death based on PIM3 calculations, PICU length of stay, need for advanced levels of critical care support, such as inhaled nitric oxide, renal replacement therapy or extracorporeal life support, did not reveal any evidence to support the hypothesis of delayed access to care. Analysis of data from several UK emergency departments confirms that delayed presentations were indeed rare [27]. The results of this study support this conclusion, although timely and appropriate public health messages from relevant public health authorities may have helped.

Overall COVID-19 and PIMS-TS accounted for fewer than 500 PICU admissions in 2020. Just under half of all reported cases of PIMS-TS in the UK and ROI were admitted to PICU and we have established that these were predominantly for vasoactive medications rather than respiratory support [2, 28]. By contrast, COVID-19 in adults resulted in excess of 25,000 critical care admissions in ICUs in England, Wales and Northern Ireland in 2020 [29]. Some of the PICU capacity freed up by the reduced number of childhood admissions were then utilised to provide critical care for the adults [5, 30]. Moreover, paediatric critical care staff were also redeployed in adult ICUs. This demonstrated an efficient and collaborative approach which was sensitive to the needs of the population, while preserving adequate paediatric critical care capacity.

Reduction in elective surgical activity was implemented in many parts of the world to free up capacity to cope with surge in COVID-19 related admissions. Several paediatric cardiac surgery programmes deferred most, if not all, elective activity [31]. This report identified the impact on elective non-cardiac surgery activity, which was higher than the observed reduction in cardiac surgical activity. These numbers provide information about the anticipated need for additional PICU capacity when elective surgeries resume. In addition, differences observed between different surgical programmes and between different regions may provide important information for future pandemic planning.

We observed important differences compared to the US report from VPS. We observed no significant differences in probability of death using PIM3, or mortality during 2020, as opposed to a small but statistically significant increase in median PIM2 and mortality in the VPS dataset. The reasons for this are not entirely clear though this UK and RoI report analysed the whole of 2020 data and is population-based.

This study reports on a well-established registry covering the whole of the UK and the RoI. Data are validated and known to be of high quality [9]. This dataset has established definitions and reports annually on numbers of admissions, severity indicators, specific diagnoses of interest and intervention related information as well as mortality. As a result we can be confident that the data we present reflect the front line experience of paediatric critical illness in the UK in 2020 and preceding years. We were able to present granular information presented in the form of monthly, as well as quarterly figures showing the evolution of the impact of the pandemic on PICU.

Nevertheless this report does have some limitations. Diagnostic category assignment is based on primary diagnosis as reported by clinicians in the participating units and some inconsistency within and between participating units may be present. Given that this data only represents designated PICUs, this data cannot be extrapolated to non-PICU hospitalisations or child deaths. A recently published study of national child mortality database in England reported that the all-cause mortality in children in 2020 (2264 deaths) was slightly lower that 2019 (2498 deaths) with a relative risk of 0.92 (0.87–0.98), *p* = 0.009) [32]. Nevertheless, comparative analysis of wider preceding time periods as well as from other countries is required before confirmation of this effect. This study does not include any other measures of health impact on children such as primary care attendances, hospitalisation, immunisation and other wider measures such as education, nutrition, fitness, poverty and mental health. These are also required to build an overall assessment to consider the risks and benefits posed by any large public health intervention in the future. An interrupted time series or regression discontinuity design analyses may be required to specifically analyse association between periods of the public health intervention measures and impact on PICU case-mix, outcomes during the pandemic. However, the timing and nature of interventions were variable at different countries. A regional approach was also pursued within some countries and these introduce significant barriers. As such our study merely describes the association between pandemic-related variables and the PICU case-mix, outcome differences, rather than test for causation.

## Conclusions

During 2020 we found a significant reduction in age–sex adjusted prevalence of unplanned PICU admissions, the number of PICU admissions, particularly secondary to respiratory diseases, and fewer child deaths in PICU. While it is possible that these may point towards a beneficial impact of population-based public health interventions during the COVID-19 pandemic, wider societal impact on physical and mental health of children are unclear. However, this could be considered as useful information, at least in part, to generate a public discourse related to the population costs and benefits of continuing adoption of public health interventions in some form or other to relieve annual winter pressures on paediatric critical care and childhood illnesses.

## Data Availability

The data that support the findings of this study are available from PICANet but restrictions apply to the availability of these data, which were used under license for the current study, and so are not publicly available. Data are, however, available from the PICANet upon reasonable request subject to PICANet’s published data access policy available on the website.
